# A Tale of Toxin Promiscuity: The Versatile Pharmacological Effects of Hcr 1b-2 Sea Anemone Peptide on Voltage-Gated Ion Channels

**DOI:** 10.3390/md20020147

**Published:** 2022-02-17

**Authors:** Ernesto Lopes Pinheiro-Junior, Rimma Kalina, Irina Gladkikh, Elena Leychenko, Jan Tytgat, Steve Peigneur

**Affiliations:** 1 Toxicology and Pharmacology, KU Leuven, O&N II Herestraat 49, P.O. Box 922, 3000 Leuven, Belgium; 2 G.B. Elyakov Pacific Institute of Bioorganic Chemistry, Far Eastern Branch, Russian Academy of Sciences, 690022 Vladivostok, Russia; kalinarimma@gmail.com (R.K.); irinagladkikh@gmail.com (I.G.); leychenko@gmail.com (E.L.)

**Keywords:** *Heteractis crispa*, APETx-like peptides, sea anemone, ion channels

## Abstract

Sea anemones are a rich source of biologically active compounds. Among approximately 1100 species described so far, *Heteractis crispa* species, also known as sebae anemone, is native to the Indo-Pacific area. As part of its venom components, the Hcr 1b-2 peptide was first described as an ASIC1a and ASIC3 inhibitor. Using *Xenopus laevis* oocytes and the two-electrode voltage-clamp technique, in the present work we describe the remarkable lack of selectivity of this toxin. Besides the acid-sensing ion channels previously described, we identified 26 new targets of this peptide, comprising 14 voltage-gated potassium channels, 9 voltage-gated sodium channels, and 3 voltage-gated calcium channels. Among them, Hcr 1b-2 is the first sea anemone peptide described to interact with isoforms from the Kv7 family and T-type Cav channels. Taken together, the diversity of Hcr 1b-2 targets turns this toxin into an interesting tool to study different types of ion channels, as well as a prototype to develop new and more specific ion channel ligands.

## 1. Introduction

Animal venoms are known as incredible libraries of active peptides, proteins, and neurotransmitters, among other components. Together, these molecules are responsible for most of the observed symptoms and hurdles during an envenomation, one of the mechanisms employed by these animals to capture and subdue their prey or predators. These toxins are a result of natural selection, in which a wide range of pharmacologically active components was carefully assorted [1,2].

Sea anemones are specimens occupying marine habitats across all depths and latitudes. They belong to the phylum Cnidaria, class Anthozoa, subclass Hexacorallia, and order Actiniaria, which comprises one of the oldest living orders of venomous species [3]. So far, there are more than 1100 species of listed sea anemones, although they remain poorly understood organisms when it comes to the investigation of active proteins and peptides, with only 5% of these species being used to isolate and characterize peptide toxins [4]. Put together, roughly 470 toxins derived from sea anemones are annotated in UniProtKB so far (https://www.uniprot.org, accessed on 10 January 2022). 

With at least 17 different structural foldings described to date, the venom of these animals represents an exceptional molecular diversity. Toxins modulating ion channels are the most abundant molecules present in these venoms [5], although actinoporins [6], Kunitz-type serine protease inhibitors [7], PLA2 [8], as well as non-proteinaceous compounds [9,10] were also reported in their arsenal.

Ion channels play a crucial role in the generation of action potentials and, consequently, in many other cellular activities, such as signal transduction, neurotransmitter release, muscular contraction, hormone secretion, cellular motility, and apoptosis [11,12]. These structures constitute the third largest group of signaling molecules encoded by the human genome [13]. Given their importance for the correct functioning of a living organism, it is not surprising that many short peptides and proteins isolated from venomous animals, such as scorpions [14], cone snails [15], spiders [16], and sea anemones, are potent and specific modulators of voltage-gated ion channels. In an earlier study [17], 3 new peptides from the hydrophobic 20% ethanol fraction from *H. crispa* venom were purified: π-AnmTX Hcr 1b-2, -3, and -4 (short names: Hcr 1b-2, -3, and -4, respectively). This fraction contains at least 159 peptide compounds, including neurotoxins and proteinase and α-amylase inhibitors, as well as modulators of ion channels. 

Hcr 1b-2, the major peptide, presents 41 residues and belongs to the class 1b of sea anemone toxins [18], with a molecular mass of 4518.9 Da (Uniprot accession number C0HL52). It was first characterized as a modulator of ASIC1a (IC_50_ 4.8 ± 0.3 μM) and ASIC3 (IC_50_ 15.9 ± 1.1 μM) channels, displaying an anti-hyperalgesic effect, reducing the pain threshold in experimental animals [17]. In this report, we expanded the electrophysiological characterization of this peptide, demonstrating that besides the activity towards ASIC channels, it also modulates 26 voltage-gated ion channels.

## 2. Results

**Figure 1:**
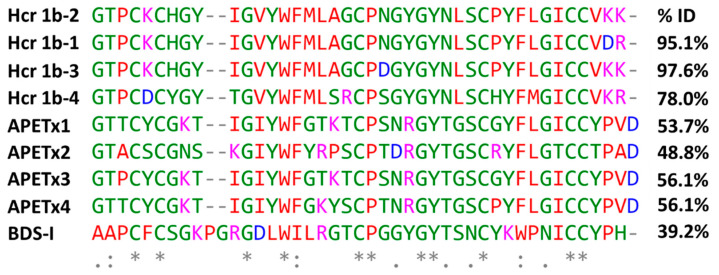
Multiple sequence alignment of Hcr 1b-2 and other peptides from *H. crispa*, peptides belonging to the APETx family, from *Anthopleura elegantissima*, and BDS-I from *Anemonia sulcate*, using the Clustal Omega alignment program. The percentages of identity (%) were obtained using the standard protein BLAST. Residues marked in red: small + hydrophobic/aromatic; blue: acidic; magenta: basic; green: hydroxyl + sulfhydryl + amine + Gly. (*) are fully conserved residues; (:) indicates conservation between groups of strongly similar properties; (.) denotes conservation between groups of weakly similar properties. Uniprot accession numbers: Hcr 1b-2: C0HL52; Hcr 1b-1: P0DL87; Hcr 1b-3: C0HL53; Hcr 1b-4: C0HL54; APETx1: P61541; APETx2: P61542; APETx3: B3EWF9; APETx4: C0HL40; BDS-I: P11494.

The purified Hcr 1b-2 peptide (Figures S1 and S2) presents sequence homology with peptides from the APETx family, isolated from the sea anemone *Anthopleura elegantissima*, and BDS-I from the sea anemone *Anemonia sulcate* (Figure 1). These peptides are known as non-selective modulators of voltage-gated potassium and sodium channels (https://www.uniprot.org, accessed on 10 January 2022). 

**Figure 2:**
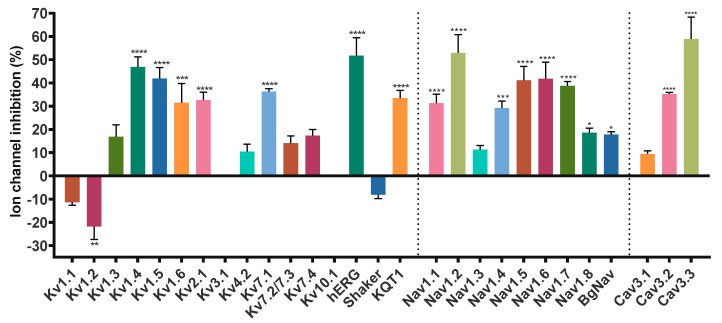
Effect of Hcr 1b-2 (1 µM) on the currents of different voltage-gated ion channels. (*n* ≥ 3) ± SEM; SEM: standard error of the mean. * *p* < 0.1; ** *p* < 0.01; *** *p* < 0.001; **** *p* < 0.0001.

Given the sequence homology, we sought to look for new potential targets of Hcr 1b-2, expanding the screening on voltage-gated potassium (K_V_), sodium (Na_V_), and calcium (Ca_V_) channels, using the two-electrode voltage-clamp technique with *Xenopus laevis* oocytes. Of the 28 screened ion channels, Hcr 1b-2 acted on 26 targets, comprising different K_V_, Na_V_, and Ca_V_ channels (Figure 2). 

### 2.1. Modulation of Kv Channels

**Figure 3:**
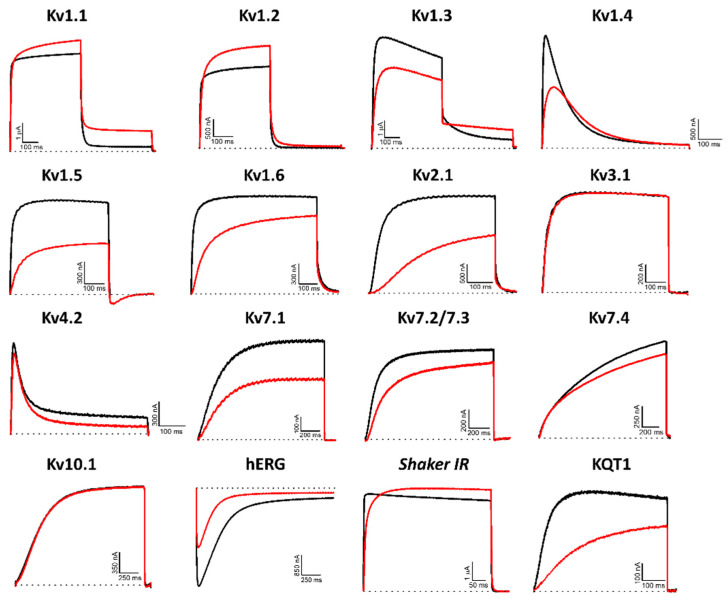
Electrophysiological characterization of Hcr 1b-2 (1 µM) on a panel of Kv channels. The black lines represent the control condition, while the red lines indicate the current obtained after the addition of the peptide. Dotted lines represent the 0 current level. The graphs illustrate the effects obtained in a series of at least three independent experiments (*n* ≥ 3). For kv1.5, the inward current seen at the tail potential at −50 mV is most likely carried by a non-selective conductance not affected by the peptide.

Hcr 1b-2 (1 µM) was screened on a panel of 14 mammalian (Kv1.1–1.6, 2.1, 3.1, 4.2, 7.1, 7.2/7.3, 7.4, 10.1, and hERG), insect (*Shaker*, from *Drosophila melanogaster*), and nematode (KQT1, from *Caenorhabditis elegans*) voltage-gated potassium channels. Among the mammalian isoforms, Hcr 1b-2 affected the activity of almost all targets, except for Kv3.1 and Kv10.1. The peptide was also capable of modulating the insect *Shaker* and the nematode KQT1 channels (Figure 3). 

The promiscuity of this peptide goes further than just acting on different Kv targets. Remarkably, both activation (Kv1.1, 1.2, and *Shaker*) and inhibition (Kv1.3, 1.4, 1.5, 1.6, 2.1, 4.2, 7.1, 7.2/7.3, 7.4, hERG, and KQT1) of potassium currents were observed. In relation to the voltage-current relationship, Hcr 1b-2 seems to have a non-significant gating-modifier effect on Kv1.1, Kv1.2, Kv1.4, Kv2.1, and Kv7.2/7.3. On the other hand, a stronger shift is observed on Kv7.1 Kv7.4, KQT1, and hERG (Table 1).

**Figure 4:**
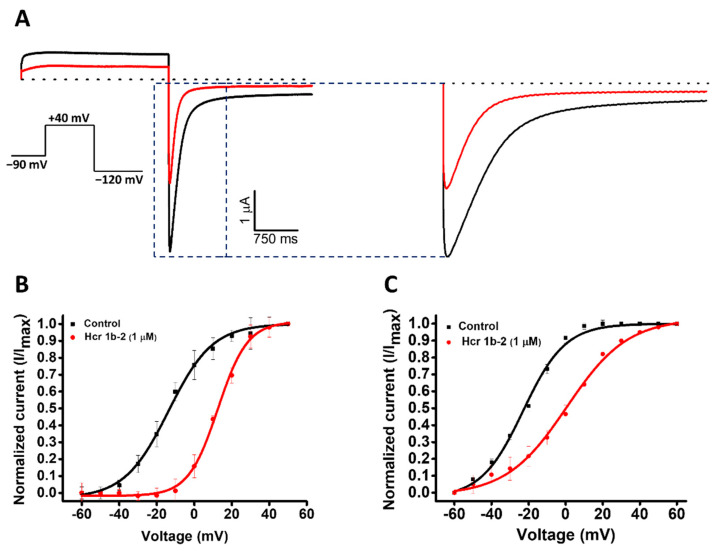
(**A**) Effect of Hcr 1b-2 (1 μM) on hERG currents. The black line represents the control condition, while the red line indicates the current obtained after the addition of the toxin. Dotted lines represent the 0 current level. The highlighted region illustrates the effect of the toxin in the tail current of this channel. The graphs illustrate the effects obtained in a series of three independent experiments (*n* = 3). The voltage-dependent effect of Hcr 1b-2 on the evoked hERG peak (**B**) and tail (**C**) currents. The normalized currents elicited in ND96 solution were plotted versus the applied pulse potentials (mV) in the control (black traces) and toxin (red traces) conditions. The data points were fitted with the Boltzmann equation.

Among the gamut of recorded activities, the most sensitive isoforms among the Kvs were hERG (51.7 ± 7.6%) and Kv1.4 (46.9 ± 4.3%), although high activities on Kv1.5 (41.9 ± 4.7%), Kv7.1 (36.2 ± 1.3%), KQT1 (33.4 ± 3.3%), Kv2.1 (32.6 ± 3.4%), and Kv1.6 (31.5 ± 6.7%) were also observed at a 1 µM concentration. Interestingly, a negligible shift in the midpoint of activation was reported for Kv1.4 (Table 1), while a strong shift towards more positive potentials was observed for hERG, both at the peak (−13.7 ± 0.5 mV to 12.6 ± 0.4 mV) and tail current (−22.9 ± 0.6 mV to 0.6 ± 0.7 mV) steps (Figure 4).

It is worth mentioning that no specificity was noticed for the activity of this peptide among different taxa. Hcr 1b-2 acted on a panel of mammalian (rat and human), insect, and nematode voltage-gated potassium channels. In the same way, the affinity was not dependent on this criteria, since distinct activities were recorded regardless of the target’s origin (Figure 3).

### 2.2. Modulation of Nav Channels

**Figure 5:**
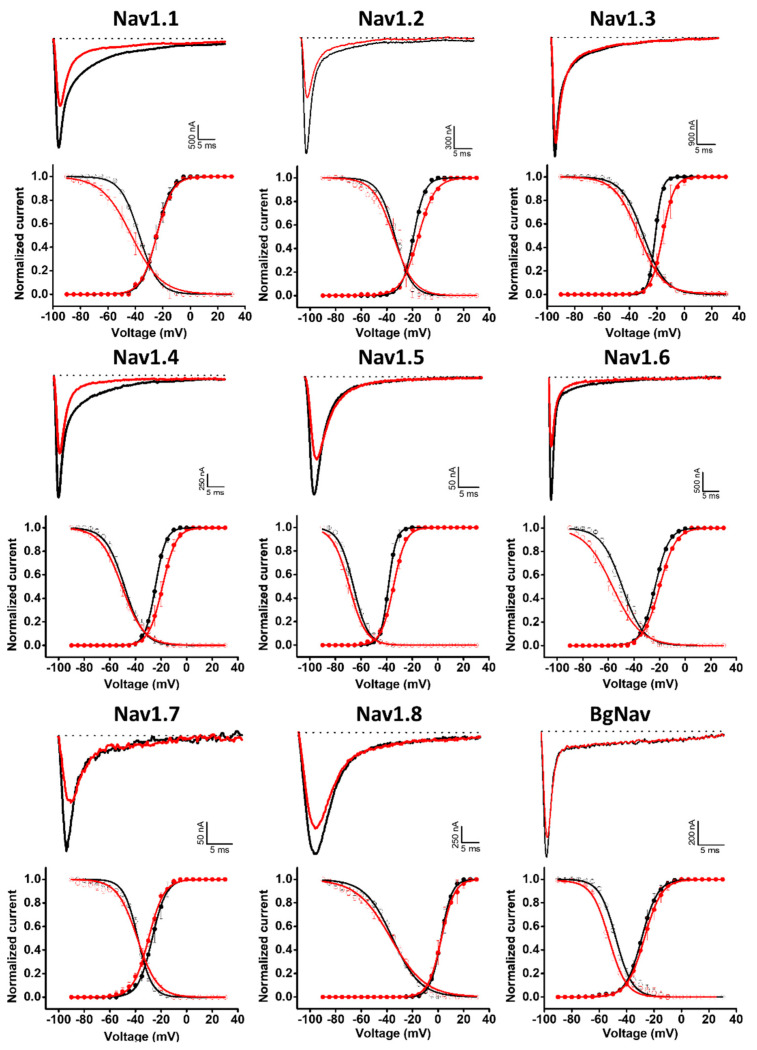
Electrophysiological characterization of Hcr 1b-2 (1 µM) on Nav channels. The upper panels represent the activity profile on several Nav channel isoforms. Representative whole-cell current traces in the control (black) and toxin (red) conditions are shown. Dotted lines indicate the 0 current level. The graphs illustrate the effects obtained in a series of at least three independent experiments (n ≥ 3). The lower panels correspond to the activation and steady-state inactivation curves in the control (black) and toxin (red) conditions. (n ≥ 3) ± SEM; SEM: standard error of the mean.

Given the similarities between the Hcr 1b-2 and APETx1–4 peptides, we also examined whether Hcr 1b-2 might have a promiscuous activity towards voltage-gated sodium channels. Remarkably, among the nine isoforms tested (mammalian Nav1.1–1.8 and BgNav, from *Blattella germanica*), Hcr 1b-2 was able to modulate all of them, at different levels. The current–voltage relationship was also assessed, in order to understand the different mechanisms by which this peptide interacts and modulates each channel isoform (Figure 5). 

Hcr 1b-2 (1 µM) had the greatest effect against the Nav1.2 isoform, in which a reduction of 52.8 ± 7.9% was observed in the peak amplitude (*I_Na_*). At the same concentration, Hcr 1b-2 also reduced the peak amplitude of Nav1.6 (41.8 ± 7.1%), Nav1.5 (41.6 ± 5.9%), Nav1.7 (38.7 ± 1.8%), Nav1.1 (31.2 ± 3.8%), Nav1.4 (29.1 ± 3.0%), Nav1.8 (18.5 ± 1.9%), and BgNav (17.7 ± 1.2%). Nav1.3 was the isoform least affected by Hcr 1b-2 since only 11.2 ± 1.8% inhibition was recorded. Notably, there is no preference of activity towards TTX-sensitive (Nav1.1 to Nav1.4, Nav1.6 and Nav1.7) and TTX-resistant (Nav1.5 and Nav1.8) channels related to Hcr 1b-2 activity.

Hcr 1b-2 also displayed different modulation mechanisms with the different tested Nav isoforms (Table 2, Figure 5). Nav1.3 and Nav1.4 activation curves were shifted towards more positive potentials in the presence of 1 µM Hcr 1b-2 (−21.2 ± 0.1 mV to −15.9 ± 0.1 mV and −24.4 ± 0.1 mV to −18.9 ± 0.1 mV, respectively). On the other hand, Hcr 1b-2 modulated the inactivation of the mammalian Nav1.1 and Nav1.6 and the insect BgNav channels towards more negative potentials (−37.2 ± 0.2 mV to −43 ± 0.4 mV, 49.1 ± 0.3 mV to −56.7 ± 0.4 mV, and −47.9 ± 0.3 to −52.9 ± 0.3, respectively). For the other isoforms (Nav1.2, Nav1.5, Nav1.7, and Nav1.8) no significant effect was observed for activation or inactivation.

### 2.3. Modulation of Cav Channels

**Figure 6:**
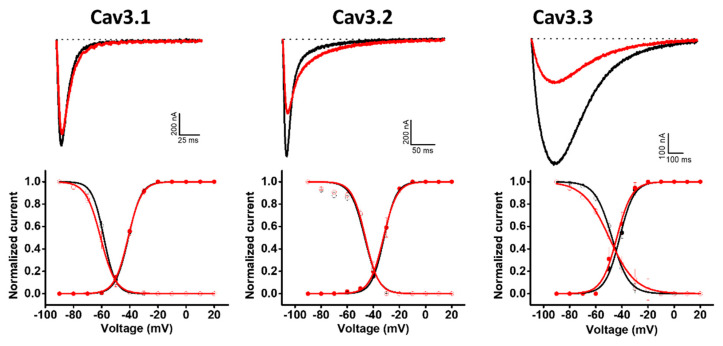
Electrophysiological characterization of Hcr 1b-2 (1 µM) on Cav channels. The upper panels represent the activity profile on three Cav channel isoforms. Representative whole-cell current traces in the control (black) and toxin (red) conditions are shown. The dotted lines indicate the 0 current level. The graphs illustrate the effects obtained in a series of at least three independent experiments (n ≥ 3). The lower panels correspond to the activation and steady-state inactivation curves in the control (black) and toxin (red) conditions. (n ≥ 3) ± SEM; SEM: standard error of the mean.

Hcr 1b-2 was also screened on a panel of three T-type voltage-gated calcium channels (Figure 6). Although the peptides belonging to the APETx family are not known as Cav channel modulators, Hcr 1b-2 (1 µM) exhibited interesting activities in this ion channel family. Among the three tested isoforms, this sea anemone toxin showed a preference towards Cav3.3, which was inhibited by 58.9 ± 9.4%, followed by Cav3.2 (35.1 ± 0.7%) and Cav1.1 (9.34 ± 1.4%).

Unlike the potassium and sodium channels, in which significant shifts in the voltage–current relationship were observed, depending on the channel isoform, Hcr 1b-2 did not show signs of modulating the voltage–sensor domains of Cav channels, acting mainly as a pore blocker. In the presence of the toxin, insignificant changes were observed in this parameter, both for activation and inactivation, when compared to the control condition (Table 3).

## 3. Discussion

Sea anemone venoms are known as rich sources of bioactive components, encompassing different types of proteins, peptides, and nonproteinaceous compounds. Although a great diversity is found, most of their components remain poorly explored [4,19]. Among the proteins and peptides, neurotoxins represent a major class of active molecules, acting on different structures, such as potassium, sodium, acid-sensing ion channels (ASIC), and other targets [3]. Given the modulation of these targets, several biological activities can be expected, encompassing autoimmune, analgesic, and central nervous system modulatory effects. 

Recently, the Hcr 1b-2 sequence was determined, together with two other peptides, named Hcr1b-3 and Hcr1b-4, which share 97.6% and 78.0% of sequence identity, respectively (Figure 1). According to previous data, these peptides were characterized as the first ASIC inhibitors derived from sea anemone venom. None of these three peptides showed signs of neurotoxicity or changes in the behavior of the tested animals [17]. 

The alignment data also shows a certain degree of similarity between Hcr 1b-2 and the toxins from the APETx family, recognized as promiscuous molecules when it comes to their targets [20]. APETx1 are known to modulate hERG [21], Nav1.2–1.6, and Nav1.8 [20], while APETx2 modulates ASICs, Kv3.4, [22], and hERG [23], as well as Nav1.2, Nav1.6, and Nav1.8 [20]. APETx3 is known for interacting with Nav1.2–1.4, Nav1.6, DmNav1, BgNav1, and VdNav1 [20]. APETx4, on the other hand, is a notorious Kv10.1 inhibitor, although it also modulates Kv1.3–1.5 and Kv2.1, as well as Nav1.4–1.6 [24]. 

In a similar way, Hcr 1b-2 targets a considerable number of the 28 ion channels detected thus far. Besides the already characterized activity on ASIC channels, here we report 26 new targets of this toxin (Figure 2), comprising several channel isoforms in different families of voltage-gated ion channels, including potassium, sodium, and calcium channels.

At 1 µM, Hcr 1b-2 could significantly shift the current-voltage relationship of hERG in a similar way as observed for APETx1 (Figure 3). Previous studies have shown that F15, Y32, F33, and L34 seem to be key residues for the interaction between APETx1 and hERG, as they are located on the molecular surface of this peptide [25]. Hcr 1b-2 shares the same residues in its sequence (Figure 1), which can explain the similar effect observed. The positive shift in the V_1/2_ value indicates that, like APETx1, Hcr 1b-2 distinguishes and stabilizes the resting state of the hERG channel, in which the voltage-sensor domain adopts the S4-down conformation. However, unlike APETx3, in which the Thr3Pro substitution abolishes the activity on this ion channel [20], Hcr 1b-2 remains a strong hERG modulator, even presenting such a residue in this position. 

Regarding the modulation of activation exerted by Hcr 1b-2 on Kv1.1, Kv1.2, and *Shaker*, only a few molecules to date are described with the same behavior. Tx7335, a three-finger toxin from *Dendroaspis angusticeps* snake venom, is capable of activating the KcsA channel present in the cellular membrane of the soil bacteria *Streptomyces lividans*. The proposed mechanism of action is the binding of this toxin to the extracellular side of the pore domain, inducing conformational changes in the channel structure and increasing its open probability [26]. This phenomenon was also observed in the interaction between charybdotoxin and the bacterial voltage-gated potassium channel KvLm [27] and between a toxin-sensitive KcsA mutant and kaliotoxin [28]. This hypothesis is also in connection with the results observed for Hcr 1b-2, since the voltage-dependency for Kv1.1 and Kv1.2 was also shifted to more negative potentials (Table 1). 

KCNQ genes encode five Kv7 channel subunits, named Kv7.1–Kv7.5. While the Kv7.1 isoform can be found in different cell lineages, including cardiac myocytes and epithelial cells [29], Kv7.2–Kv7.5 are mainly found in the nervous system, as Kv7.2 and Kv7.3 are the main molecular components of the slow voltage-gated M-channel responsible for fine-tuning neuronal excitability [30]. In respect to the already characterized modulators of this voltage-gated potassium channel family, only a few examples of animal-derived components are currently known.

The scorpion toxin ImKTx104, from *Isometrus maculatus* venom, was the first peptide characterized as a Kv7.1 inhibitor, with a K_d_ of 11.69 µM [31]. To date, Hcr 1b-2 is the second animal toxin known to interact with this channel isoform. Moreover, AaTXKβ_2–64_, a peptide from *Androctonus australis* scorpion venom, activates the isoforms Kv7.2/7.3, Kv7.5/7.3, Kv7.3, and Kv7.4, whereas the Kv7.1 and Kv7.2 homomeric channels were not affected. Especially regarding Kv7.4, this peptide caused a shift of 10 mV towards more negative potentials [32]. 

In an opposite direction, Hcr 1b-2 inhibits the Kv7.1, Kv7.2/7.3, and Kv7.4 isoforms. To the best of our knowledge, Hcr 1b-2 is the first known toxin from a sea anemone species able to inhibit the homomeric Kv7.1 and Kv7.4 channels, as well as the heteromeric Kv7.2/7.3 channel. This finding opens new paths and perspectives to understand how toxins were naturally designed to act on these particular targets, as well as to assist the development of novel molecules capable of interacting with them.

Analyzing the voltage–current relationship for this Kv family (Table 1), no significant shift was observed for Kv7.2/7.3 in the presence of the toxin. Contrariwise, a shift towards more positive potentials was observed for Kv7.1, Kv7.4, and for the KCNQ-like channel (KQT1) from *Caenorhabditis elegans* (Table 1). Based on these outcomes, we hypothesize that Hcr 1b-2 interacts at different sites of the channel structure, depending on the Kv7 isoform. The same is proposed for the Nav channels, discussed later.

APETx1 and APETx2 are well-known sea anemone peptides that are characterized as sodium channel modulators. Like Hcr 1b-2, APETx1 inhibits the mammalian Nav1.2–1.6 and Nav1.8 isoforms [20], while APETx2 acts on Nav1.2, Nav1.6, and Nav1.8. Hcr 1b-2 also displayed inhibitory activities on Nav1.1 (68.7 ± 3.8%), Nav1.7 (38.7 ± 1.8%), and the insect channel BgNav (17.5 ± 1.2%), isoforms that are unaffected by APETx1 and APETx2. Additionally, while APETx2 did not modulate the voltage-dependence of Nav channels, Hcr 1b-2 affected both the activation (Nav1.3 and Nav1.4) and inactivation (Nav1.1, Nav1.6, and BgNav) processes. Nonetheless, further studies are required to understand the mechanisms by which Hcr 1b-2 causes different effects depending on the Nav isoform.

So far, sea anemone toxins, including peptides belonging to the APETx family, were well-described promiscuous molecules, interacting mostly with voltage-gated potassium and sodium channels. The indiscrimination of targets observed for Hcr 1b-2 goes further, affecting the T-type Cav3.1, Cav3.2, and Cav3.3 channels. Additionally, a discrimination between the tested isoforms was also noted, given the preference towards Cav3.3, with a 9-fold and 1.6-fold higher activity than the ones measured on Cav3.1 and Cav3.2, respectively. However, unlike the effects shown on Kv and Nav channels, there was no significant shift in the activation and inactivation processes. 

The only targets in which Hcr 1b-2 was not active were Kv3.1 and Kv10.1 (Figure 2 and Figure 3). Among sea anemone toxins, BDS-I, from *Anemonia sulcata*, is a well-characterized toxin capable of inhibiting isoforms from the Kv3 family, being active on Kv3.1, Kv3.2, and Kv3.4 [33]. It also acts on neuronal sodium channels, however, unlike Hcr 1b-2, it strongly enhances TTX-sensitive channels, while TTX-resistant isoforms are weakly affected [34]. 

On the other hand, Kv10.1 is a complex target and only a few toxins were found to date capable of interacting with this channel. One of the reasons lies in its extracellular structure, which presents two putative glycosylation sites (Asp388 and Asp406) [35]. These sugar chains surround the pore region and may prevent the binding of inhibitory toxins [36]. As previously discussed, APETx4 is the most known non-selective Kv10.1 inhibitor toxin from a sea anemone. Besides APETx4, collinein-1, a snake venom serine protease from *Crotalus durissus collilineatus*, is another marvelous Kv10.1 inhibitor, presenting a high specificity and a mechanism independent of its enzymatic activity [37]. 

## 4. Materials and Methods

### 4.1. Peptide Isolation and Primary Structure Determination

The specimens of *H. crispa* were collected from the South China Sea, Vietnam (2013), frozen, and kept at −20 °C. The Hcr 1b-2 was isolated from the 70% water-ethanol extract of *H. crispa* whole body by hydrophobic chromatography on a Polychrome-1 column (4.8 × 95 cm) (Olaine, Latvia) in step gradient of ethanol concentration and RP-HPLC on a Luna C18 column and an analytical Nucleosil C18 column using a step gradient of acetonitrile (with 0.1% TFA). The first 39 amino acids from the N-terminal sequence of alkylated Hcr 1b-2 were determined using automated Edman sequencing with a Procise 492 cLC protein sequencing system (Applied Biosystems, Waltham, MA, USA). Two C-terminal residues were identified with a mass spectrometer MaXis impact (Bruker Daltonik, Karlsruhe, Germany) from the collision-induced dissociation tandem mass spectra of two peptide fragments obtained by the cyanogen bromide cleavage of the 4-vinylpyridine-treated Hcr 1b-2 [17].

### 4.2. Electrophysiological Assays

#### 4.2.1. Expression of Ion Channels in Xenopus Laevis Oocytes

For the expression of Kv channels (rKv1.1, rKv1.2, rKv1.3, rKv1.4, rK1.5, rKv1.6, hKv2.1, hKv3.1, rKv4.2, hKv7.1, hKv7.2/7.3, hKv7.4, KQT1 (from the roundworm *Caenorhabditis elegans*), hKv10.1 (hEAG1), hKv11.1 (hERG1), and *Shaker*, from *Drosophila melanogaster*), Nav channels (rNav1.1, rNav1.2, rNav1.3, rNav1.4, hNav1.5, mNav1.6, hNav1.7, hNav1.8, and BgNav (from the cockroach *Blattella germanica*), as well as the hβ1 and rβ1 subunits, and Cav channels (rCav3.1, hCav3.2, and rCav3.3) in *Xenopus* oocytes, the linearized plasmids were transcribed using the T7 or SP6 mMESSAGEmMACHINE transcription kit (Ambion, Austin, TX, USA). Mature female animals were purchased from Nasco (Fort Atkinson, WI, USA) and were housed in the Aquatic Facility (KU Leuven) in compliance with the regulations of the European Union (EU) concerning the welfare of laboratory animals as declared in Directive 2010/63/EU. The use of *X. laevis* oocytes was approved by the Animal Ethics Committee of the KU Leuven, with the license number P186/2019. 

Stage V–VI oocytes were collected from anesthetized female *X. laevis* frog as previously described [38,39], with the frogs anesthetized by placement in a 0.1% tricaine solution (amino benzoic acid ethyl ester; Merck, Kenilworth, NJ, USA). Oocyte microinjection was performed using a microinjector (Drummond Scientifc^®^, Broomall, PA, USA), with a programmed cRNA injection volume of 4–50 nL, depending on channel subtype. The oocytes were incubated in ND96 solution (96 mM NaCl; 2 mM KCl; 1.8 mM CaCl_2_; 2 mM MgCl_2_, and 5 mM HEPES, pH 7.4), supplemented with 50 mg/L of gentamicin sulfate. 

#### 4.2.2. Electrophysiological Recordings

Electrophysiological measurements were performed at room temperature (18–22 °C) using the two-electrode voltage-clamp (TEVC) technique. Data were obtained using a GeneClamp 500 amplifier (Axon Instruments^®^, Burlingame, CA, USA) and Clampex9 software (Axon Instruments^®^, USA) was responsible for data acquisition and storage. Glass micropipettes were produced using glass capillaries (borosilicate WPI 1B120-6) and drawn in a WPI (World Precision Instruments^®^, Sarasota, FL, USA) manual stretcher. The bath and perfusion solutions were either the previously described ND96 (Nav and Kv channels) or calcium-free ND96 supplemented with 10 mM BaCl_2_ (Cav channels).

Whole-cell currents of oocytes were recorded 1 to 3 days after RNA injection. The current and voltage electrodes were filled with 3 M KCl, and their resistance was adjusted from 1.0 to 2.5 MΩ. Currents were sampled at 20 kHz (Nav channels) and 10 kHz (Kv and Cav channels) and filtered using a four-pole Bessel low-pass Bessel filter at 1 kHz for sodium and 500 MHz for potassium and calcium, except for the hERG ion channel in which the currents were filtered at 1 kHz. Leak subtraction was performed using a P/4 protocol. Kv1.x currents were evoked by 500 ms depolarizations to 0 mV, followed by a 500 ms pulse to −50 mV from a holding potential of −90 mV. Kv2.1, Kv3.1, Kv4.2, Kv7.x, and KQT1 currents were elicited by 700 ms pulses to +20 mV from a holding potential of −90 mV. Current traces of the hERG1 channel were elicited by applying a +40 mV prepulse for 2 s, followed by a step of −120 mV for 2 s. Current traces of hEAG1 were elicited by a 2 s depolarization to 0 mV from a holding potential of −90 mV. Sodium current traces were evoked by a 100 ms depolarization to 0 mV. For Cav channels, current traces were elicited by 700 ms depolarizations to −20 mV from a holding potential of −90 mV. The current–voltage relationships were determined by 50 ms step depolarizations between −90 and +65 mV using 5 or 10 mV increments. All electrophysiological data were analyzed following the procedures described by Peigneur et al., 2012 [20].

All values were expressed as means ± SEM. Differences in ionic currents between the control and toxin conditions were compared by a one-way ANOVA, followed by Dunnett’s multiple comparisons test. Differences were considered statistically significant when *p* < 0.1.

## 5. Conclusions

Given all the activities presented by Hcr 1b-2, this toxin can be used as a tool to study different types of ion channels, as well as to be employed as a prototype aiming at the development of novel and more specific ion channel ligands. Nonetheless, further studies are needed to expand our understanding in how this peptide interacts with its targets on a molecular level. Moreover, we discovered the first sea anemone venom peptide capable of interacting with the Kv7 and T-type Cav channels, broadening the perspectives for studying the interaction of animal toxins with such structures.

Collectively, our findings demonstrate that the Hcr 1b-2 peptide from *H. crispa* venom displays an exceptional lack of selectivity. It is worth mentioning that this molecule presents a relatively small size, with only 41 amino acids carefully arranged in a way to present this incredible dynamism among its targets. From the 28 potential ligands, comprising 16 voltage-gated potassium channels, 9 voltage-gated sodium channels, and 3 voltage-gated calcium channels, 26 of them were subject to a certain degree of activation or inhibition by this toxin. Although the mechanism of action of this peptide on each target, as well as its structural features and effects *in vivo*, remain to be further explored, these results shed new light on how nature evolves to design molecules capable of acting on so many different structures. 

## Figures and Tables

**Table 1:** Shifts in the current–voltage relationship in the activation of voltage-gated potassium channels in control and in the presence of 1 µM Hcr 1b-2.

	V_half_ Activation (mV ± SEM)		
Channel Isoform	Control (ND96)	Hcr 1b-2 (1 µM)	Shift (mV)
Kv1.1	−13.8 ± 0.3	−18.6 ± 0.3	−4.8
Kv1.2	−24.7 ± 0.7	−29.6 ± 0.89	−4.9
Kv1.4	7.5 ± 0.7	10.0 ± 0.5	2.5
Kv2.1	5.3 ± 0.3	8.0 ± 0.2	2.6
Kv7.1	5.8 ± 0.4	12.2 ± 0.6	6.4
Kv7.2/7.3	14.6 ± 0.5	15.9 ± 0.7	1.3
Kv7.4	3.8 ± 0.3	10.9 ± 0.3	7.1
KQT1	14.6 ± 0.4	22.4 ± 0.5	7.9
hERG (peak)	−13.7 ± 0.5	12.6 ± 0.4	25.7
hERG (tail)	−22.9 ± 0.6	0.6 ± 0.7	24.8

**Table 2:** Shifts in current–voltage relationships of voltage-gated sodium channels in control and in the presence of 1 µM Hcr 1b-2.

	V_half_ Activation (mV ± SEM)			V_half_ Inactivation (mV ± SEM)		
Channel Isoform	Control (ND96)	Hcr 1b-2 (1 µM)	Shift (mV)	Control (ND96)	Hcr 1b-2 (1 µM)	Shift (mV)
**Nav1.1**	−24.6 ± 0.1	−24.5 ± 0.1	0.1	−37.2 ± 0.2	−43 ± 0.4	−5.8
**Nav1.2**	−19.2 ± 0.1	−15.5 ± 0.2	3.7	−33.6 ± 0.4	−34.7 ± 0.6	−1.1
**Nav1.3**	−21.2 ± 0.1	−15.9 ± 0.1	5.3	−29.9 ± 0.1	−33.3 ± 0.1	−3.4
**Nav1.4**	−24.4 ± 0.1	−18.9 ± 0.1	5.5	−48.5 ± 0.3	−50.2 ± 0.2	−1.7
**Nav1.5**	−38.5 ± 0.1	−34.7 ± 0.1	3.8	−66.0 ± 0.2	−69.2 ± 0.2	−3.2
**Nav1.6**	−23.5 ± 0.1	−20.0 ± 0.1	3.5	−49.1 ± 0.3	−56.7 ± 0.4	−7.6
**Nav1.7**	−26.9 ± 0.1	−29.7 ± 0.1	−2.8	−38.1 ± 0.3	−38.6 ± 0.4	−0.5
**Nav1.8**	−2.3 ± 0.1	−2.6 ± 0.1	−0.3	−35.6 ± 0.4	−36.9 ± 0.5	−1.3
**BgNav**	−29.0 ± 0.1	−27.1 ± 0.1	1.9	−47.9 ± 0.3	−52.9 ± 0.3	−5.0

**Table 3:** Shifts in current–voltage relationships of voltage-gated calcium channels in control and in the presence of 1 µM Hcr 1b-2.

	V_half_ Activation (mV)			V_half_ Inactivation (mV)		
Channel Isoform	Control (ND96 Ca^2+^ Free)	Hcr 1b-2 (1 µM)	Shift	Control (ND96 Ca^2+^ Free)	Hcr 1b-2 (1 µM)	Shift
**Cav3.1**	−41.0 ± 0.1	−41.2 ± 0.1	−0.2	−58.4 ± 0.2	−60.7 ± 0.2	−2.3
**Cav3.2**	−31.9 ± 0.1	−32.3 ± 0.2	−0.4	−46.6 ± 0.8	−46.1 ± 0.6	0.5
**Cav3.3**	−41.7 ± 0.4	−43.9 ± 0.5	−2.2	−47.6 ± 0.2	−49.7 ± 0.3	−2.1

## Data Availability

The data presented in this study are available on request from the corresponding author. The data are not publicly available due to privacy.

## Supplementary Materials

The following are available online at https://www.mdpi.com/article/10.3390/md20020147/s1, Figure S1: RP-HPLC performed on a Luna C18 column (10 × 250 mm, Phenomenex, Torrance, CA, USA), equilibrated with 10% acetonitrile solution in 0.1% trifluoroacetic acid (TFA) on an Agilent 1100 chromatograph (Agilent Technologies, Santa Clara, CA, USA). Elution was carried out in a linear gradient of acetonitrile concentration from 0 to 70% in 0.1 % trifluoroacetic acid, for 70 minutes. Flow rate: 1.5 mL/min, Figure S2: ESI mass spectrum of Hcr 1b-2. Calculated average and monoisotopic molecular masses are 4522.3206 and 4519.0042 Da, measured 4521.9614 and 4518.9564 Da, respectively.
