# Supplementary material for: A Tale of Toxin Promiscuity: The Versatile Pharmacological Effects of Hcr 1b-2 Sea Anemone Peptide on Voltage-Gated Ion Channels
Source: Mar Drugs. 2022 Feb 17;20(2):147. doi: 10.3390/md20020147 (PMC8878452; doi:10.3390/md20020147)
Supplement: Supplementary file 1 [file marinedrugs-20-00147-s001.zip › marinedrugs-1575821-supplementary.pdf]

Article

A tale of toxin promiscuity: The versatile pharmacological effects of Hcr 1b-2 sea anemone peptide on voltage-gated ion channels

Ernesto Lopes Pinheiro-Junior[1]*, Rimma Kalina[2], Irina Gladkikh[2], Elena Leychenko[2], Jan Tytgat[1]*, Steve Peigneur[1]*

Supplementary material

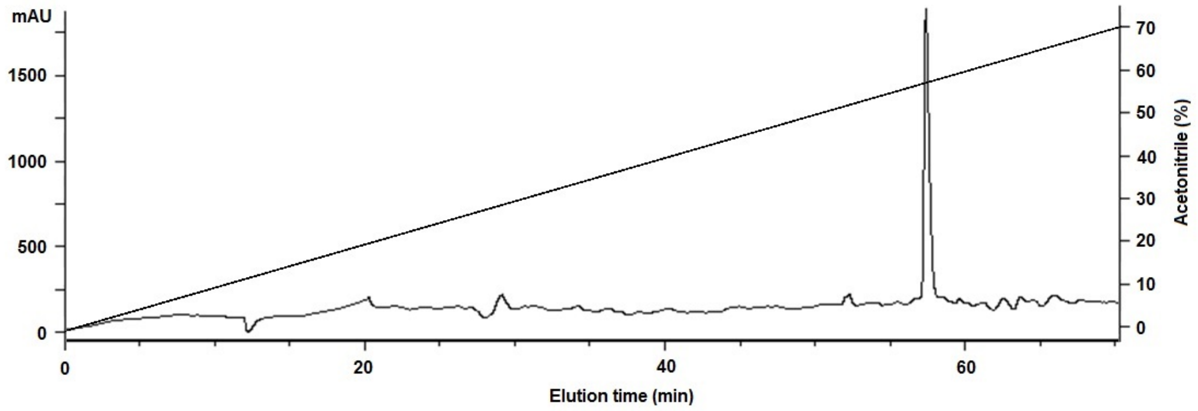

Figure S1. RP-HPLC performed on a Luna C18 column (10 × 250 mm, Phenomenex, USA), equilibrated with 10% acetonitrile solution in 0.1% trifluoroacetic acid (TFA) on an Agilent 1100 chromatograph (Agilent Technologies, USA). Elution was carried out in a linear gradient of acetonitrile concentration from 0 to 70% in 0.1 % trifluoroacetic acid, for 70 minutes. Flow rate: 1.5 mL/min.

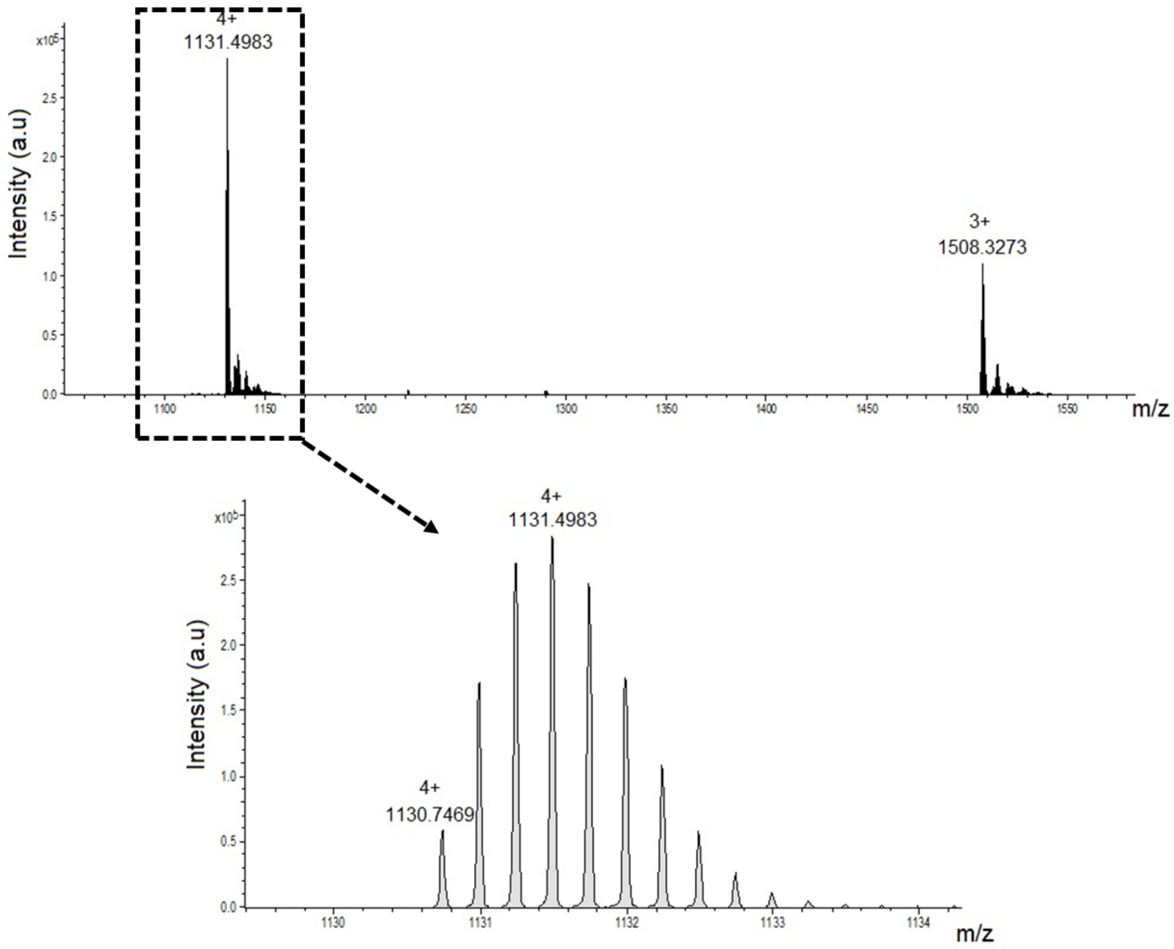

Figure S2. ESI mass spectrum of Hcr 1b-2. Calculated average and monoisotopic molecular masses are 4522.3206 and 4519.0042 Da, measured 4521.9614 and 4518.9564 Da, respectively.
